# Impact of Cost-Related Medication Nonadherence on Economic Burdens, Productivity Loss, and Functional Abilities: Management of Cancer Survivors in Medicare

**DOI:** 10.3389/fphar.2021.706289

**Published:** 2021-06-29

**Authors:** Z. Kevin Lu, Xiaomo Xiong, Jacob Brown, Ashley Horras, Jing Yuan, Minghui Li

**Affiliations:** ^1^ Department of Clinical Pharmacy and Outcomes Sciences, College of Pharmacy, University of South Carolina, Columbia, SC, United States; ^2^ Department of Clinical Pharmacy and Pharmacy Administration, School of Pharmacy, Fudan University, Shanghai, China; ^3^ Department of Clinical Pharmacy and Translational Science, University of Tennessee Health Science Center, Memphis, TN, United States

**Keywords:** cost-related medication nonadherence, economic burdens, productivity loss, functional abilities, cancer survivors

## Abstract

**Background:** Cancer survivors are vulnerable to have medication nonadherence. We aimed to estimate the impact of cost-related medication nonadherence on economic burdens, productivity loss, and functional abilities among cancer survivors.

**Methods:** A cross-sectional study was conducted using data from the National Health Interview Survey (NHIS), 2011–2018. Cost-related medication nonadherence was identified based on NHIS prompts. An ordinal logistic regression model was used to determine the impact of cost-related medication nonadherence on survivors’ economic burden. Two negative binomial regression models were implemented to estimate the impact on productivity loss. In addition, four logistic regression models were used to determine the impact on functional abilities. The weighted analysis was used to generate national estimates.

**Results:** Among 35, 773, 286 cancer survivors, 15, 002, 192 (41.9%) respondents reported that they experienced cost-related medication nonadherence. Compared to cancer survivors without cost-related medication nonadherence, those with nonadherence were significantly associated with an increased economic burden (OR: 1.89, 95% CI: 1.70–2.11). Also, cancer survivors with cost-related medication nonadherence were significantly more likely to have an increased bed disability day (IRR: 1.46, 95% CI: 1.21–1.76). In terms of the limitations, cancer survivors with nonadherence were significantly more likely to have both activity limitation (OR: 1.42, 95% CI: 1.25–1.60) and functional limitation (OR: 2.12, 95% CI: 1.81–2.49).

**Conclusion:** Cost-related medication nonadherence increased economic burdens, productivity loss, and limitations in functional abilities among cancer survivors. Strategies are needed to help cancer survivors with cost-related medication nonadherence to be adherent to prescriptions.

## Background

An estimated 16.9 million cancer survivors were living in the United States in 2019 (American Cancer Society, 2019). Due to the potential increase in the size of the population, the number of cancer survivors is estimated to increase to 22.1 million in 2030 (American Cancer Society, 2019). Cancer survivors are often treated with extensive and expensive treatments such as chemotherapy, immunotherapy, and nonpharmacological treatments (Baskar et al., 2012). Since cancer survivors are more likely to have chronic comorbidities, they have to be treated for those chronic illnesses as well (Mirza et al., 2018). Due to the high costs of the medications in the treatment and the treatment itself, cancer survivors are more than twice as likely to not adhere to medication treatments (Zhang and Meltzer, 2015; Smith et al., 2019; Zhao et al., 2019).

With the development of innovative interventions for cancer, the death rate of cancer has been decreasing continuously (Siegel et al., 2019), but cancer survivors consequently face a heavier economic burden due to the treatment in their extended life years (Carlotto et al., 2013). Evidence shows that the medical costs for cancer care had increased annually regardless of the type of cancer from 2010 to 2017 in the United States and the total medical costs of cancer care are projected to increase to $157.8 billion by 2020 (Mariotto et al., 2011; Guy et al., 2017). For cancer survivors, although medication nonadherence might decrease pharmacy costs, it could significantly increase other costs, such as hospital costs and indirect costs (Cutler et al., 2018). Cost-related medication nonadherence to cancer medications has been shown to increase the total healthcare costs (Nekhlyudov et al., 2011; Kaul et al., 2017). Given an increased likelihood of comorbidities caused by medication nonadherence, costs for other chronic conditions may increase (Cutler et al., 2018). Cancer survivors with cost-related medication nonadherence are more likely to have a worse quality of life due to the disease progression led by insufficient but necessary health care (Meneses et al., 2012; Fenn et al., 2014). Under the situation of insufficient health care and lower quality of life, cancer survivors may lose more productivity and have a worse condition of functional abilities (Bouwman et al., 2017; Martin et al., 2018; Chou et al., 2019).

However, little is known about the association between cost-related medication nonadherence and economic burdens among cancer survivors. No literature has shown the potential impact of cost-related medication nonadherence on cancer survivors’ productivity loss and functional abilities. This study used a retrospective pooled cross-sectional study with the National Health Interview Survey (NHIS), a large nationally representative cohort study of United States adults, to study the impact of cost-related medication nonadherence on economic burdens, productivity loss, and functional abilities among cancer survivors.

## Methods

### Data and Study Population

This is a retrospective pooled cross-sectional study using the data from the NHIS, 2011–2018. The NHIS is an ongoing, national, long-term, cross-regional, annual family interview survey of the civilian non-institutionalized United States population, held annually by the National Center for Health Statistics (NCHS) of the Centers for disease Control and Prevention (CDC) (National Center for Healt, 2020). NHIS sample is designed and weighted to be representative of the United States population, using a multistage probability sample design (National Center for Healt, 2020). The detailed sampling and survey methods of NHIS are provided elsewhere (Parsons et al., 2014). We used NHIS because it collected information on cancer survivors annually.

The period of 2011–2018 was used because the information on cost-related medication nonadherence started being reported in 2011 and 2018 is the latest data available. We included cancer survivors aged 18 years or older. To be consistent with previous studies on cancer survivors using NHIS, we excluded cancer survivors with only nonmelanoma skin cancers (Greenlee et al., 2016; Boyd et al., 2020; Dee et al., 2020). Also, we excluded cancer survivors who had an unknown or missing value on NHIS prompts of cost-related medication nonadherence.

### Measures

Cancer survivors were identified if they have ever been told by a doctor or other health professional that they had cancer. Cost-related medication nonadherence was defined as the failure to make the required prescriptions due to costs, and cancer survivors with cost-related medication adherence were determined if they had answered “yes” to any of the following prompts: “During the past 12 months, in order to save money, did you 1) delay refilling prescription? 2) Take less medication? 3) Skipped medication doses?” The economic burden was measured as the amount of family health care spending in the past 12 months, which was categorized into four levels ($0, $1-$1,999, $2,000–4,999, $5,000 or more). Productivity loss was measured as the work-loss days and bed disability days of the survivors in the past 12 months. Limitations included activity limitation, functional limitation, activities of daily living (ADL) limitation, and instrumental activities of daily living (IADL) limitation. Specifically, ADL refers to an individual’s daily self-care activities, while IADL indicates daily activities that require more complex interactions (Katz, 1983). Activity and functional limitations were determined if respondents answered “yes” to the relevant prompts asking whether they had the limitation in the past 12 months. ADL limitation was identified if respondents answered “yes” to an NHIS prompt asking whether they needed help with daily self-care activities, such as eating, bathing, dressing, or getting around inside the house. IADL limitation was identified if respondents answered “yes” to an NHIS prompt asking whether they needed help in handling routine needs, such as everyday household chores, doing necessary business, shopping, or getting around for other purposes.

Five demographic variables were included as covariates: age (18–29, 30–44, 45–64, and ≥65), gender (male and female), race (non-Hispanic White, non-Hispanic Black, Hispanic, and others), marital status (single and non-single), and census region (Northeast, North, Central/Midwest, South, and West); three socioeconomic variables were included as covariates: education attainment (below high school, high school, above high school), family income (<$50,000, $50,000–$99,999, ≥$100,000), and health care insurance (yes and no); and two physical health-related variables: body mass index (BMI) (<18.5, 18.5–24.9, 25–29.9, and ≥30) and general health status (good/very good/excellent, fair/poor).

### Statistical Analysis

The chi-square and *t*-test were used to compare the baseline characteristics between cancer survivors who reported cost-related medication nonadherence and those who did not. The prevalence of cost-related medication adherence was measured using the number of respondents who reported cost-related medication nonadherence divided by the total number of respondents. Logistic regression models were used to identify if there was a significant trend of the cost-related medication nonadherence by using the prevalence as the dependent variable and the year as the independent variable. We performed an ordinal logistic regression model to examine the impact of cost-related medication nonadherence on economic burdens, two negative binomial regression models for the impact on productivity loss, and four logistic regression models for the impact on limitations. The results of the logistic regression models were reported as odds ratios (OR) with 95% confidence intervals (CI), while the results of the negative binomial regression models were reported as incidence rate ratios (IRR) with 95% confidence intervals (CI).

All analyses were performed using SAS 9.4 software (SAS Institute Inc., Cary, NC). NHIS-constructed survey weights were applied to account for the NHIS complex stratified sampling methods and to make estimates that are representative of the United States civilian non-institutionalized population.

## Results

**FIGURE 1:**
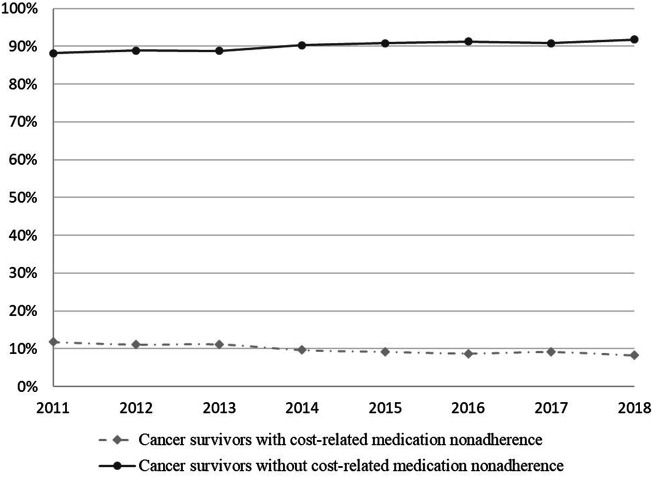
Trend of cost-related medication nonadherence among cancer survivors between 2011 and 2018.

**FIGURE 2:**
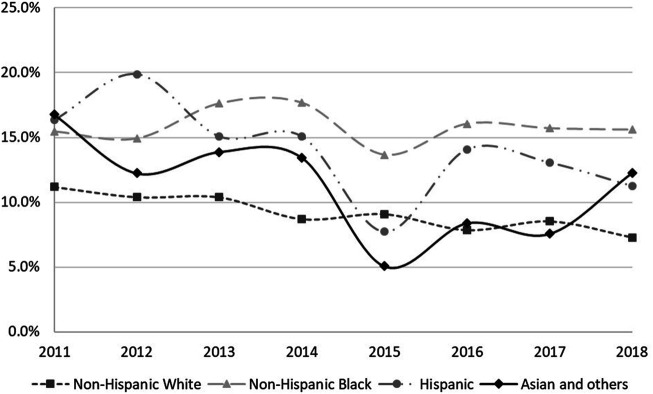
Racial/ethnic disparities in cost-related medication nonadherence among cancer survivors between 2011 and 2018.

Among 22,908 cancer survivors, 2,302 (10.0%) respondents reported that they had cost-related medication nonadherence in the past 12 months. After weighting, among 75, 690, 823 cancer survivors, 7,464,168 (9.8%) reported cost-related medication nonadherence. There was a decreasing trend of cost-related medication nonadherence among cancer survivors between 2011 and 2018 (*p* < 0.001, Figure 1). The trend by race and ethnicity showed that among cancer survivors, only the trend in non-Hispanic Whites was continuously decreasing, while there was no specific pattern of the trends among other races (Figure 2). Compared to non-Hispanic Whites, non-Hispanic Blacks and Hispanics reported a higher prevalence of cost-related medication nonadherence.

Table 1 demonstrated the comparisons of baseline characteristics between cancer survivors with or without cost-related medication nonadherence. All characteristics were significantly different between cancer survivors with or without cost-related medication nonadherence. Compared with cancer survivors without cost-related medication nonadherence, those with nonadherence were more likely to have a higher economic burden (*p* < 0.001), more activity limitation (*p* < 0.001) and more functional limitation (*p* < 0.001), as well as more likely to need the help of ADL (*p* < 0.001) and IADL (*p* < 0.001). In addition, cancer survivors with cost-related medication nonadherence had longer average work-loss days (*p* < 0.001) and bed disability days (*p* < 0.001).

**TABLE 1:** Baseline characteristics of cancer survivors with or without cost-related medication nonadherence.

Characteristics	With cost-related medication nonadherence (*n* = 2,302)	Without cost-related medication nonadherence (*n* = 20,606)	*P*-Value
	Sample No. (weighted %),Mean (SE)	Sample No. (weighted %),Mean (SE)	
Year			<0.001
2011	348 (12.4%)	2494 (15.2%)	
2012	369 (12.4%)	2679 (14.2%)	
2013	306 (11.2%)	2370 (12.9%)	
2014	292 (11.3%)	2684 (11.1%)	
2015	258 (12.1%)	2617 (11.2%)	
2016	272 (13.1%)	2953 (11.5%)	
2017	244 (13.2%)	2431 (12.2%)	
2018	213 (14.2%)	2378 (11.7%)	
Age			<0.001
≥18 and <30	92 (1.4%)	278 (3.8%)	
≥30 and <45	334 (5.4%)	1110 (14.4%)	
≥45 and <65	1157 (30.1%)	6141 (50.6%)	
≥65	719 (63.1%)	13077 (31.3%)	
Gender			<0.001
Female	650 (29.2%)	8665 (41.9%)	
Male	1652 (70.8%)	11941 (58.1%)	
Marital status			<0.001
Married	814 (36.2%)	9827 (48.3%)	
Non-married	1484 (63.8%)	10742 (51.7%)	
Race/ethnicity			<0.001
Non-hispanic white	1756 (79.6%)	17341 (86.6%)	
Non-hispanic black	287 (10.8%)	1541 (6.3%)	
Hispanic	173 (6.4%)	1036 (4.4%)	
Other^a^	86 (3.1%)	688 (2.8%)	
Living region			<0.001
Northeast	286 (13.3%)	3659 (18.3%)	
North central/Midwest	525 (25.4%)	4831 (24.3%)	
South	942 (41.1%)	7182 (36.5%)	
West	549 (20.2%)	4934 (20.9%)	
Education			<0.001
Below high school	378 (14.7%)	2688 (12.1%)	
High school graduate	597 (26.6%)	5318 (25.4%)	
Above high school	1327 (58.7%)	12600 (62.5%)	
Family income			<0.001
< $50,000	1659 (73.3%)	10147 (51.2%)	
≥ $50,000 and < $100,000	414 (19.6%)	5277 (28.4%)	
≥ $100,000	139 (7.1%)	3574 (20.4%)	
Health status			<0.001
At least good	1092 (24.3%)	5176 (46.5%)	
Poor or fair	1209 (75.7%)	15414 (53.5%)	
BMI			<0.001
<18.5	56 (2.2%)	408 (1.9%)	
≥18.5 and <25	574 (25.7%)	6359 (31.3%)	
≥25 and <30	669 (29.3%)	7245 (35.4%)	
≥30	1003 (42.8%)	6594 (31.5%)	
Health insurance			<0.001
No	927 (39.1%)	6296 (30.4%)	
Yes	1374 (60.9%)	14279 (69.6%)	
Family healthcare spending			<0.001
$0	127 (5.2%)	1664 (8%)	
> $0 and < $2,000	1323 (57.9%)	12891 (63.5%)	
≥ $2,000 and < $5,000	525 (23.9%)	3754 (19%)	
≥ $5,000	297 (13%)	1801 (9.5%)	
Activity limitation			<0.001
No	1004 (44.4%)	12962 (63.5%)	
Yes	1296 (55.6%)	7635 (36.5%)	
Function limitation			<0.001
No	372 (16.3%)	6777 (33.4%)	
Yes	1929 (83.7%)	13813 (66.6%)	
ADL limitation			<0.001
No	2108 (92.1%)	19376 (94.1%)	
Yes	194 (7.9%)	1228 (5.9%)	
IADL limitation			<0.001
No	1904 (83.4%)	18114 (88.2%)	
Yes	398 (16.6%)	2487 (11.8%)	
Work-loss days	8.08 (0.37)	13.04 (1.28)	<0.001
Bed disability days	8.35 (0.28)	24.31 (1.52)	<0.001

ADL: activities of daily living; BMI: body mass index; IADL: instrumental activities of daily living; SE: Standard error.

a Other includes Asian, Aleut, Alaskan Native, or American Indian, Pacific Islander, Hawaiian, Samoan, Guamanian, and multiple races.

In the multivariable analysis, we found that compared to cancer survivors without cost-related medication nonadherence, those with the nonadherence were associated with a higher economic burden (OR: 1.89, 95% CI: 1.70–2.11) (Table 2). Cancer survivors with cost-related medication nonadherence were more likely to have an increased bed disability day (IRR: 1.46, 95% CI: 1.21–1.76) (Table 2). However, the association between cost-related medication nonadherence and the increased work-loss day was not significant. In terms of functional abilities, cancer survivors with nonadherence were more likely to have both activity limitation (OR: 1.42, 95% CI: 1.25–1.60) and functional limitation (OR: 2.12, 95% CI: 1.81–2.49) (Table 3). The association of cost-related medication nonadherence with either ADL limitation (OR: 0.90, 95% CI: 0.73–1.11) or IADL limitation (OR: 0.98, 95% CI: 0.83–1.16) was not significant.

**TABLE 2:** The impact of cost-related medication nonadherence on economic burdens and productivity loss among cancer survivors.

Variables	Economic burdens, OR (95% CI)^a^	Work-loss days, IRR (95%CI)^a^	Bed disability days, IRR (95% CI)^a^
Cost-related medication nonadherence			
No	Ref		
Yes	1.89 (1.70–2.11)	1.28 (0.92–1.78)	1.46 (1.21–1.76)
Age			
≥18 and <30	Ref		
≥30 and <45	1.58 (1.16–2.16)	1.93 (1.28–2.92)	1.38 (0.92–2.07)
≥45 and <65	1.84 (1.36–2.49)	1.33 (0.95–1.87)	1.03 (0.73–1.47)
≥65	1.52 (1.13–2.05)	0.90 (0.59–1.37)	0.66 (0.46–0.95)
Sex			
Female	Ref		
Male	0.99 (0.92–1.05)	1.20 (0.98–1.47)	1.72 (1.43–2.05)
Marital status			
Married	Ref		
Non-married	0.53 (0.50–0.57)	0.91 (0.74–1.11)	0.84 (0.71–1.00)
Race/ethnicity			
Non-hispanic white	Ref		
Non-hispanic black	0.50 (0.44–0.57)	1.54 (0.89–2.64)	1.32 (0.95–1.82)
Hispanic	0.69 (0.58–0.82)	1.14 (0.75–1.74)	1.52 (1.07–2.16)
Other^b^	0.66 (0.53–0.82)	1.37 (0.67–2.80)	1.20 (0.90–1.61)
Living region			
Northeast	Ref		
North central/Midwest	1.17 (1.06–1.29)	0.90 (0.68–1.19)	0.92 (0.65–1.29)
South	1.22 (1.11–1.33)	1.11 (0.84–1.45)	0.95 (0.69–1.30)
West	1.18 (1.07–1.32)	1.24 (0.91–1.68)	0.98 (0.71–1.37)
Education			
Below high school	Ref		
High school graduate	1.29 (1.15–1.44)	1.23 (0.78–1.95)	0.95 (0.69–1.30)
Above high school	1.68 (1.50–1.88)	0.91 (0.59–1.41)	0.90 (0.67–1.22)
Family income			
< $50,000	Ref		
≥ $50,000 and < $100,000	1.93 (1.78–2.09)	0.98 (0.76–1.27)	0.77 (0.63–0.93)
≥ $100,000	2.62 (2.36–2.91)	0.82 (0.61–1.11)	0.80 (0.59–1.08)
Health insurance			
No	Ref		
Yes	1.05 (0.98–1.13)	1.35 (1.00–1.82)	0.95 (0.76–1.19)
BMI			
<18.5	Ref		
≥18.5 and <25	0.97 (0.76–1.25)	1.65 (0.80–3.39)	1.51 (0.94–2.42)
≥25 and <30	0.96 (0.89–1.04)	0.93 (0.72–1.20)	0.89 (0.70–1.11)
≥30	0.98 (0.90–1.06)	1.03 (0.79–1.34)	0.90 (0.74–1.11)
Health status			
At least good	Ref		
Poor or fair	1.15 (1.11–1.19)	1.85 (1.66–2.06)	2.29 (2.13–2.46)

BMI: body mass index; CI: Confidence interval; OR: Odds ratio.

a The results are weighted.

b Other includes Asian, Aleut, Alaskan Native, or American Indian, Pacific Islander, Hawaiian, Samoan, Guamanian, and multiple races.

**TABLE 3:** The impact of cost-related medication nonadherence on functional abilities among cancer survivors.

Variables	Activity limitation, OR (95% CI)^a^	Functional limitation, OR (95% CI)^a^	ADL limitation, OR (95%CI)^a^	IADL limitation, OR (95%CI)^a^
Cost-related medication nonadherence				
No	Ref	Ref	Ref	Ref
Yes	1.42 (1.25–1.60)	2.12 (1.81–2.49)	0.90 (0.73–1.11)	0.98 (0.83–1.16)
Age				
18–29	Ref	Ref	Ref	Ref
30–44	1.83 (1.23–2.72)	1.15 (0.82–1.63)	1.25 (0.49–3.20)	1.42 (0.73–2.78)
45–64	2.73 (1.88–3.95)	2.41 (1.73–3.36)	1.86 (0.76–4.58)	1.79 (0.97–3.29)
≥65	3.57 (2.46–5.18)	4.89 (3.52–6.81)	3.39 (1.38–8.31)	3.11 (1.70–5.70)
Gender				
Female	Ref	Ref	Ref	Ref
Male	0.93 (0.86–1.01)	1.54 (1.42–1.66)	1.26 (1.09–1.45)	1.38 (1.24–1.54)
Marital status				
Married	Ref	Ref	Ref	Ref
Non-married	1.57 (1.45–1.70)	1.13 (1.03–1.23)	1.41 (1.20–1.65)	2.02 (1.78–2.29)
Race				
Non-hispanic white	Ref	Ref	Ref	Ref
Non-hispanic black	0.91 (0.80–1.04)	0.82 (0.70–0.96)	1.15 (0.94–1.41)	0.94 (0.80–1.12)
Hispanic	0.76 (0.64–0.90)	0.77 (0.64–0.92)	1.42 (1.10–1.83)	0.96 (0.76–1.21)
Other^b^	0.94 (0.75–1.17)	0.76 (0.61–0.94)	1.61 (1.15–2.25)	1.48 (1.14–1.91)
Living region				
Northeast	Ref	Ref	Ref	Ref
North central/Midwest	1.13 (1.00–1.28)	1.22 (1.08–1.39)	0.87 (0.69–1.10)	1.00 (0.84–1.20)
South	1.02 (0.90–1.15)	1.10 (0.98–1.24)	0.88 (0.72–1.07)	0.84 (0.72–0.98)
West	1.28 (1.12–1.45)	1.17 (1.03–1.33)	1.06 (0.85–1.33)	1.06 (0.89–1.25)
Education				
Below high school	Ref	Ref	Ref	Ref
High school graduate	0.82 (0.73–0.92)	0.90 (0.77–1.05)	0.86 (0.70–1.05)	0.92 (0.79–1.07)
Above high school	0.86 (0.76–0.97)	0.85 (0.74–0.97)	0.77 (0.63–0.95)	0.94 (0.81–1.09)
Family income				
< $50,000	Ref	Ref	Ref	Ref
$50,000–$99,999	0.57 (0.52–0.63)	0.78 (0.70–0.86)	0.80 (0.66–0.98)	0.66 (0.57–0.77)
≥ $100,000	0.41 (0.36–0.47)	0.63 (0.56–0.71)	0.68 (0.50–0.93)	0.60 (0.48–0.75)
Health insurance				
No	Ref	Ref	Ref	Ref
Yes	0.91 (0.84–0.99)	0.97 (0.89–1.06)	1.14 (0.97–1.33)	1.06 (0.94–1.19)
BMI				
18.5–24.9	Ref	Ref	Ref	Ref
<18.5	1.63 (1.22–2.18)	1.26 (0.93–1.72)	1.89 (1.35–2.64)	2.09 (1.50–2.90)
25–29.9	0.97 (0.88–1.07)	1.34 (1.22–1.47)	0.82 (0.68–0.99)	0.87 (0.76–1.01)
≥30	1.22 (1.11–1.34)	2.07 (1.88–2.28)	1.02 (0.86–1.22)	1.04 (0.91–1.18)
Health status				
At least good	Ref	Ref	Ref	Ref
Poor or fair	2.76 (2.64–2.89)	2.25 (2.16–2.34)	3.06 (2.78–3.37)	2.72 (2.54–2.90)

ADL: activities of daily living; CI: Confidence interval; IADL: instrumental activities of daily living; OR: Odds ratio.

a The results are weighted.

b Other includes Asian, Aleut, Alaskan Native, or American Indian, Pacific Islander, Hawaiian, Samoan, Guamanian, and multiple races.

## Discussion

Using a nationally representative dataset, our study examined the impact of cost-related medication nonadherence on economic burdens and productivity as well as activity limitation and functional limitation among cancer survivors. We found that there was a significant decreasing trend in the self-reported cost-related medication nonadherence throughout the study period. Also, the results showed that cost-related medication nonadherence is significantly associated with increased economic burdens and productivity loss. Cancer survivors were more likely to have worse functional abilities.

The significant decreasing trend of cost-related medication nonadherence to health care might be because of the Patient Protection and Affordable Care Act (ACA), which was implemented in 2011, and the majority of substantial changes were nationally implemented in 2014 that was covered by the period of our study (Dresden et al., 2017). With the enaction of ACA, more affordable health insurance plans were aimed to be established, thereby lowering the total medical costs for low-income households (French et al., 2016; Dresden et al., 2017). In addition, ACA encouraged the expansion of Medicaid program coverage to allow more adults in most states to be financially capable of receiving their treatment with less stress regarding billing issues (Mechanic, 2014). Such measures outlined in ACA might help gradually reduce the prevalence of cost-related medication nonadherence among cancer survivors (Dresden et al., 2017).

Contrary to the purpose of saving money, cost-related medication nonadherence led to an unintended consequence that was associated with an increased amount of family health care costs, which was likely due to a number of varying factors. Evidence has shown that medication nonadherence is associated with an increase in complications of chronic conditions and hospital visits (Kaul et al., 2017). Also, medication nonadherence is associated with the progression of the disease for cancer survivors, which is likely to worsen the quality of life of patients (Shafrin et al., 2017; Zhou et al., 2019). Given an increase in the amount of annual health care utilization and a worse quality of life, it is likely there would be an increase in the costs of patients (Epstein et al., 2017; Sawaya et al., 2019). Similar results were also observed in previous studies. It is known that low adherence to medications could lead to increased indirect costs in cardiovascular patients (Bansilal et al., 2015). Similarly, it has been found that nonadherent patients with bipolar disorder have increased indirect costs compared to adherent cohorts (Bagalman et al., 2010).

Increased productivity loss is another significantly associated outcome with cost-related medication nonadherence. In comparison with those without cost-related medication nonadherence, respondents with nonadherence were 28 and 46% more likely to have an increased work-loss day and bed disability day, respectively. Similar to economic burdens, more work-loss days and bed disability days could be associated with the poor clinical outcome of medication nonadherence. This is likely because of the fact that medication nonadherence would cause a worse clinical outcome in cancer survivors, which could further force them to take more “sick” days in comparison to adherent survivors (Chung et al., 2019). As mentioned above, medication nonadherence has shown to worsen the disease condition and to increase hospitalizations, thus, work-loss days and bed disability days would increase (Kaul et al., 2017).

Cost-related medication nonadherence in cancer survivors is also associated with activity limitation and functional limitation. Therefore, emphasizing cost-related adherence can help improve later quality of life outcomes and decrease the likelihood of limitations in everyday life. Healthcare providers and pharmacists could help in explaining the activity limitation and functional limitation that could occur with cost-related medication nonadherence. Providing patients with this information could shepherd them towards continuously and strictly following the guidelines of their prescriptions in an effort to avoid possible limitations and decreased quality of life.

It is important to emphasize medication adherence to cancer survivors. The results of this research can be used to highlight the importance of medication adherence for cancer survivors. When trying to prevent direct and indirect costs in relation to cost-related medication nonadherence, strategies to teach and inform cancer survivors of the financial importance of adhering to medication regimens can be extremely important in easing the possible long-term financial burden that is associated with cancer treatment. Healthcare providers could help implement strategies or interventions that educate a patient on the possible financial outcomes of cost-related medication nonadherence not only will improve a cancer survivors’ financial situation, but also will improve the clinical outcome. Policymakers and third-party payers can help teach medication adherence to patients because it can help both of them save money. Since medication nonadherence is shown to increase hospitalizations and other healthcare visits, third-party payers like Medicare and Medicaid, as well as other policymakers, can highlight the savings related to medication adherence for cancer survivors, as it can also help save the third-party payers and policymakers money. In the end, there will always be financial burdens due to the high costs of cancer medications source from the background, so there should be a strong effort to decrease medication costs in order to improve medication adherence of both cancer survivors and other patients dealing with chronic illnesses.

To our knowledge, this study is the largest, most contemporary population-based analysis of the impact of cost-related medication nonadherence on economic burdens, productivity loss, and functional abilities among cancer survivors. Also, we used a nationally representative dataset to generalized the results to a national level. However, this study has several limitations. First, the measurement of dependent and independent variables was based on a self-report survey, which might cause recall biases in the results to a certain extent given that cancer survivors with worse functional abilities might have memory impairment or confusion when responding to survey questions. However, considering that productivity loss and limitations cannot be captured by claims data, survey data was the best source for the measurement of the outcomes in this study. Second, the survey did not do subgroup analyses for different cancer types. Cancer survivors with different types might have a different prevalence of cost-related medication nonadherence, and the impact of cost-related medication nonadherence might be different. Finally, because we used a cross-sectional study design, no conclusions on the casual inference between cost-related medication nonadherence and outcomes could be drawn.

## Conclusion

Cancer survivors with cost-related medication nonadherence are more likely to have a higher economic burden and to have more productivity loss. In addition, cost-related medication nonadherence is associated with an increased probability of having activity limitation and functional limitation. The results highlight the need to draw increased attention to cost-related medication nonadherence among cancer survivors. Strategies are needed to help cancer survivors with cost-related medication nonadherence to be more adherent to prescriptions.

## Data Availability

Publicly available datasets were analyzed in this study. This data can be found here: https://www.cdc.gov/nchs/nhis/index.htm.
